# Supplementary material for: A Fyn biosensor reveals pulsatile, spatially localized kinase activity and signaling crosstalk in live mammalian cells
Source: eLife. 2020 Feb 4;9:e50571. doi: 10.7554/eLife.50571 (PMC7000222; doi:10.7554/eLife.50571)
Supplement: Supplementary file 1. [file elife-50571-supp1.docx]

**Supplementary File 1A.** Structural Statistics of Fyn-SH3/F29 complex.

|  | **Fyn-SH3/F29** |
| --- | --- |
| ***Restraints*** |  |
| Unambiguous restraints | 06 |
| Ambiguous restraints | 16 |
|  |  |
| ***Haddock Statistics*** |  |
| Cluster Size | 200 |
| Haddock score | -98.1(± 14.4) |
| Van Der Waals Energy | -56.9 (± 11.6) |
| Electrostatic Energy | -235.4 (± 43.2) |
| Non-bonded energy | -292.2 (± 42.6) |
| Surface area | +1436.7 (± 104.9) |
|  |  |
| ***Rmsd*** |  |
| Bond angles | 0.5 |
| Bond lengths | 0.003 |
| *Rmsd^a^* |  |
| All backbone | 0.5 |
| All heavy atoms | 0.7 |
| MolprobityClashscore^b^ | 1.53 |
|  |  |
| ***Ramachandran Map*** |  |
| Most Favoured regions | 94.7 % |
| Allowed regions | 5.2 % |
| Disallowed regions | 0.1 % |

^a^Calculated for an ensemble of 10 lowest energy structures.

^b^Calculated for the lowest energy structure.

**Supplementary File 1B.** Interactions observed in the Fyn-SH3:F29 complex

| **Fyn-SH3** | | | **F29** | | |
| --- | --- | --- | --- | --- | --- |
| Hydrophobic Interactions within 5A° | | | | | |
| Residue  Position | Residue  type | Atom | Residue  Position | Residue  type | Atom |
| 90 | LEU |  | **26*** | TYR |  |
| 91 | TYR |  | **26** | TYR |  |
| 119 | TRP |  | **22** | PHE |  |
| 119 | TRP |  | **31** | PHE |  |
| 119 | TRP |  | **43** | PHE |  |
| 132 | TYR |  | **43** | PHE |  |
| 134 | PRO |  | **31** | PHE |  |
| 137 | TYR |  | **26** | TYR |  |
| Side-chain: Main-chain hydrogen bonds | | | | | |
| 96 | ARG | NH1 | **21** | VAL | O |
| 96 | ARG | NH1 | **21** | VAL | O |
| 96 | ARG | NE | 64 | LYS | O |
| 119 | TRP | NE1 | 32 | PHE | O |
| 136 | ASN | ND2 | **24** | THR | O |
| 136 | ASN | ND2 | **24** | THR | O |
| 136 | ASN | ND2 | **29** | GLN | O |
| 136 | ASN | ND2 | **29** | GLN | O |
| 137 | TYR | OH | 25 | ARG | O |
| 116 | GLU | O | 8 | TYR | OH |
| Side-chain: Side-chain hydrogen bonds | | | | | |
| 118 | ASP | OD1 | 9 | LYS | NZ |
| 93 | TYR | OH | **24** | THR | OG1 |
| 99 | ASP | OD1 | **33** | ARG | NH1 |
| 99 | ASP | OD1 | **33** | ARG | NH1 |
| Aromatic Interactions | | | | | |
| 91 | TYR |  | **22** | PHE |  |
| 119 | TRP |  | **26** | TYR |  |
| 119 | TRP |  | **43** | PHE |  |
| 132 | TYR |  | **43** | PHE |  |
| 137 | TYR |  | **26** | TYR |  |

*The residues that are mutated from Sso7d (original scaffold) to F29 are marked in bold
