# Supplementary material for: A Fyn biosensor reveals pulsatile, spatially localized kinase activity and signaling crosstalk in live mammalian cells
Source: eLife. 2020 Feb 4;9:e50571. doi: 10.7554/eLife.50571 (PMC7000222; doi:10.7554/eLife.50571)
Supplement: Supplementary file 2. [file elife-50571-supp2.docx]

**Supplementary File 2A.** List of constructs used in study.

| **Name** | **Gene of Interest** | **Modifications in constructs** | **Product of fusion protein** | **Vector and Hosts** |
| --- | --- | --- | --- | --- |
| p-SUMO-ppx-FynSH3 | FynSH3 domain | Thrombin cleavage site (LVPRGSH) was replaced with Pre-Scission protease cleavage site (LEVLFQGP) | 6X-His-SUMO-ppx-FynSH3 | pET14b, Bacteria |
| p-SUMO-ppx-Src SH3 | Src SH3 domain |  | 6X-His-SUMO-ppx-Src SH3 | pET14b, Bacteria |
| p-SUMO-ppx-F29 | F29, Fyn SH3 binder |  | 6X-His-SUMO-ppx-F29 | pET14b, Bacteria |
| p-SUMO-ppx-Src SH3 T99D | Src SH3 domain mutant |  | 6X-His-SUMO-ppx-Src SH3 T99D | pET14b, Bacteria |
| p-GST-ppx-L_15_-F29 | F29, Fyn SH3 binder | Pre-Scission protease cleavage site and F29 ORF was fused with gene flexible linker (GGGGS)3 | GST-ppx-L_15_-F29 | pGEX6P1, Bacteria |
| p-GST-ppx-L_15_-F29 P41A | Fyn Binder point mutant |  | GST-ppx-L_15_-F29 P41A | pGX6P1, Bacteria |
| p-GST-ppx-L_15_-F29 R33A |  |  | GST-ppx-L_15_-F29 R33A | pGX6P1, Bacteria |
| p-GST-ppx-Fyn SH3 | Fyn SH3 domain | none | GST-ppx-Fyn SH3 | pGX6P1, Bacteria |
| p-GST-ppx-Src SH3 | Src SH3 domain | none | GST-ppx-Src SH3 | pGX6P1, Bacteria |
| p-GST-ppx-Yes SH3 | Yes SH3 domain | none | GST-ppx-Yes SH3 | pGX6P1, Bacteria |
| p-FynWT | h-FynWT | none | Fyn Kinase WT | pTriex4neo, Dual |
| p-FynCA | h-FynCA Y531F |  | Fyn kinase CA (Y531F) | pTriex4neo, Dual |
| p-UD-L_15_-mCerulean-L_15_-FynWT | Labeled h-FynWT | mCerulean was inserted between Ud and SH3 domain of Fyn flanked by linker (GGGGS)3 | UD-L_15_-mCerulean-L_15_-FynWT | pTriex4neo, Dual |
| p-UD-L_15_-mCerulean-L_15_-FynCA | Labeled h-FynCA |  | UD-L_15_-mCerulean-L_15_-FynCA Y531F | pTriex4neo, Dual |
| p-FynWT- L_15_-mCerulean | Labeled h-FynWT | mCerulean was fused after Fyn flanked by flexible linker (GGGGS)3 | Fyn- L_15_-mCerulean WT | pTriex4neo, Dual |
| p-mCerulean | mCerulean | none | mCerulean | pTriex4neo, Dual |
| p-mVenus | mVenus | none | mVenus | pTriex4neo, Dual |
| p-mVenus-L_15_-F29 | FynSH3 domain binder | mVenus and Binder ORF was fused with flexible linker (GGGGS)3 | mVenus-L_15_-F29 | pTriex4neo, Dual |
| p-mVenus-L_15_-F29 P41A |  |  | mVenus-L_15_-F29 P41A | pTriex4neo, Dual |
| p-*myr*-mVenus-L_15_-F29 |  | myristoyl group (MGSSKSKPKDPS) was fused before mVenus | *myr*-mVenus-L_15_-F29 | pTriex4neo, Dual |
| p-*myr*–mVenus-L_15_-F29 P41A |  |  | *myr*-mVenus-L_15_-F29 P41A | pTriex4neo, Dual |
| p-*myr*-mVenus | mVenus |  | *myr*-mVenus | pTriex4neo, Dual |

**Supplementary File 2B.** Lists of primers used in study.

| **SN** | **Name** | **Sequence (5’-----3’)** | **Specifications** |
| --- | --- | --- | --- |
| **1** | P2R | GGGCCCCTGGAACAGAACTTCCAGACCGGAACTTGCCGCCGCGTACG | Underline is overlapping sequence of prescission protease cleavage site (LEVLFQGP) |
| **2** | P3F-Fyn | CTGGAAGTTCTGTTCCAGGGGCCCATGTTCGTTGCTCTGTATG |  |
| **3** | P3F-Src | CTGGAAGTTCTGTTCCAGGGGCCCATGACCTTCGTCGCCCTGTATGAC |  |
| **4** | Src SH3 T99D FP | CGTACCGAAGATGATCTGAGCTTTAAAAAAGGTGAACGCC | Underline region is codon for aspartic acid |
| **5** | Src SH3 T99D RP | GCTCAGATCATCTTCGGTACGGGATTCATAG |  |
| **6** | FB Nde FP | GGCTCGAGCATATGGCGACCGTGAAAT | Underline are restriction sites for *Nde*I and *Bam*H1 |
| **7** | FB BamH1 RP | AAGCGGATCCTTATTTTTTCTGTTTTTCC |  |
| **8** | pGEXPS15L FP | CTGTTCCAGGGGCCCCTG*GGATCC*GGTGGAGGCGGTTCAGGCGGAG | Underlines are overlap regions with pGEX6P1. *Bam*H1 and *Xho*I site are shown in italics. |
| **9** | F29 pGEX RP | GCGGCCG*CTCGAG*TCGACCTTATTTTTTCTGTTTTTCCAGCTTCTGCAGCAG |  |
| **10** | F29 P41A FP | ATCTGGGCGGCGGCAAAGCGGGCTTTGGCGTCGTGAGCGA | Underline is codon for alanine |
| **11** | F29 P41A FP | CGCCAAAGCCCGCTTTGCCGCCGCCCAGATCATATCTAAA |  |
| **12** | F29 R33A FP | AGCGATTTTT TTTGCGTATG ATCTGGGCGG CGGC |  |
| **13** | F29 R33A RP | CCCAGATCATACGCAAAAAAAATCGCTTTGCCGT |  |
| **14** | 15L-BC-FynSH3 FP | GGCGGAGGTGGCTCT*GGCGGTGGCGGATCG*ACAGGAGTGACACTCTTTGTGGCCC | Yellow highlighted are coding sequence of flexible linker. Underlined region are for B, italic for C and regular for A |
| **15** | UD-15L-AB RP | AGAGCCACCTCCGCCTGAACCGCCTCCACCTCCTCCTCTCGTACGCAAGGTCCCCG |  |
| **16** | 15L-BC-CER-VEN FP | GGCGGAGGTGGCTCT*GGCGGTGGCGGATCG*ATGGTGAGCAAGGGCGAGGAGCTG |  |
| **17** | CER-VEN 15L AB RP | AGAGCCACCTCCGCCTGAACCGCCTCCACCCTTGTACAGCTCGTCCATGCCGAG |  |
| **18** | FynY527F FP | CAGAGCCCCAGTTCCAACCTGGTGAAAACC | Underline is codon for Phenylalanine |
| **19** | FynY527F RP | CACCAGGTTGGAACTGGGGCTCTGTCGCGG |  |
| **20** | UdFynNcoI FP | CAAAGGAGATATACCATGGGCTGTGTGCAATGTAAGG | Underline are restriction sites for *Nco*I and *Bam*H1 enzymes |
| **21** | FynBamH1 RP | CTGAGAATTCGGATTCTTACAGGTTTTCACCAGGTTGGTACTGGGGCTC |  |
| **22** | CER-VEN NcoI FP | CAAAGGAGATATACCATGGTGAGCAAGGGCGAGGAGCTG | Underline is *Nco*I site |
| **23** | CER-VEN BamH1 RP | CTGAGAATTCGGATTCTTATTGTACAGCTCGTCCATGCCGAG | Underline is *Bam*H1 site |
| **24** | 15L-BC-F29 FP | GGCGGAGGTGGCTCT*GGCGGTGGCGGATCG*ATGGCGACCGTGAAATTTAAATATAAAGGCG | Same for SN 18 primer |
| **25** | F29 BamH1 RP | GCCGAGATCTGAGAATTCGGATCCTTATTTTTTCTGTTTTTCCAGCTTCTGCAGCAG | Underline BamH1 site |
| **26** | Myr CER-VEN FP | ATGGGCAGTAGCAAGAGCAAGCCTAAGGACCCCAGC ATGGTGAGCAAGGGCGAGGAG | Grey highlighted are coding sequence of myristoyl group tag |
| **27** | PTriexMyr RP | GCTGGGGTCCTTAGGCTTGCTCTTGCTACTGCCCATGGTATATCTCCTTTGATTGTAAATAAAATGTAATTACAGTATAG |  |
