# Supplementary material for: A Fyn biosensor reveals pulsatile, spatially localized kinase activity and signaling crosstalk in live mammalian cells
Source: eLife. 2020 Feb 4;9:e50571. doi: 10.7554/eLife.50571 (PMC7000222; doi:10.7554/eLife.50571)
Supplement: Supplementary file 3. [file elife-50571-supp3.docx]

**Supplementary File 3:**

| **Key Resources Table** | | | | |
| --- | --- | --- | --- | --- |
| **Reagent type (species) or resource** | **Designation** | **Source or reference** | **Identifiers** | **Additional information** |
| strain, strain background (*E. coli*) | *E. coli* BL21(DE3) | New England Bio Labs | Cat # C2527I | Competent *E. coli* |
| strain, strain background (*E. coli*) | *E. coli*  XL10-Gold | Agilent Technologies | Cat # 200314 | Ultracompetent *E. coli* |
| genetic reagent (yeast surface display) | Randomized Sso7d scaffold library in Yeast Surface display platform | B.M. Rao Laboratory, North Carolina State University, Raleigh, North Carolina, USA | ([Gera et al., 2013](#_ENREF_1)) | Used for Fyn SH3 binder screening |
| cell line (*Homo sapiens)* | Human Embryonic Kidney Cells (HEK-293T | Jyotsna Dhawan laboratory (inStem, India) | ([Saleh et al., 2019](#_ENREF_2)) |  |
| cell line (*Homo sapiens)* | Human Bone Osteosarcoma Epithelial Cells line (U2OS) | Satyajit Mayor (NCBS, India) Laboratory | ([Kalappurakkal et al., 2019](#_ENREF_2)) |  |
| cell line (*Mus musculus)* | C2C12 mouse muscle myoblasts cell line) | American Type Culture Collection USA (ATCC) | cat # CRL-1772, lot #70013341 |  |
| transfected  construct (*Homo sapiens*) | AmphoPack-293 cell line | Dr Reety Arora Jyotsna Dhawan laboratory (inStem, India) |  | Used as host for retrovirus production only |
| antibody | anti-Myc Tag (Chicken polyclonal) | Thermo Fisher Scientific | CAT# A-21281, RRID:AB_2535826 | Flow (1:250) |
| antibody | anti-Chicken IgY (H+L)  Cross-Adsorbed Secondary Antibody,  Alexa Fluor 633 (Goat polyclonal) | Thermo Fisher Scientific | CAT# A-21103  RRID:AB_2535756 | Flow (1:250) |
| antibody | anti-Fyn (Rabbit polyclonal) | Cell Signaling Technology | Cat# 4023, RRID:AB_10698604 | WB (1:750) |
| antibody | anti-Fyn (Mouse monoclonal) | Abcam | Cat# ab3116, RRID:AB_303524 | IF (1:250) |
| antibody | anti- Phospho-Src Family-Tyr416  Rabbit polyclonal) | Cell Signaling Technology | Cat# 2101, RRID:AB_331697 | WB (1:750) |
| antibody | anti- beta-Actin (Rabbit polyclonal) | Cell Signaling Technology | Cat# 4967, RRID:AB_330288 | WB (1:1000) |
| antibody | anti-GFP (Rabbit polyclonal) | Abcam | Cat# ab290, RRID:AB_303395 | WB (1:5000) |
| antibody | Anti- GFP (D5.1) XP (Rabbit monoclonal) | Cell Signaling Technology | Cat# 2956, RRID:AB_1196615 | WB (1:1000) |
| antibody | anti-poly-Histidine Unconjugated clone HIS-1 (Mouse monoclonal) | Sigma-Aldrich | Cat# H1029, RRID:AB_260015 | WB (1:3000) |
| antibody | anti-mouse IgG, HRP-linked (Horse unknown) | Cell Signaling Technology | Cat# 7076, RRID:AB_330924 | WB (1:3000) |
| antibody | anti-rabbit IgG, HRP-linked (Goat polyclonal) | Cell Signaling Technology | Cat# 7074, RRID:AB_2099233 | WB (1:3000) |
| antibody | Anti p44/42 MAPKErk1/2, 137F5 (Rabbit monoclonal) | Cell Signaling Technology | Cat# 4695, RRID:AB_390779 | WB (1:1000) |
| antibody | anti-Phospho-p44/42 MAPK Erk1/2, Thr202/Tyr204 (Rabbit polyclonal) | Cell Signaling Technology | Cat# 9101, RRID:AB_331646 | WB (1:1000) |
| antibody | anti-Vinculin  (Mouse monoclonal) | Sigma-Aldrich | Cat# V9131, RRID:AB_477629 | WB (1:500) |
| antibody | anti-YES1  (Rabbit polyclonal) | Sigma-Aldrich | Cat# HPA026480, RRID:AB_1858921 | WB (1:200) |
| antibody | anti-SRC  (Rabbit polyclonal) | Sigma-Aldrich | Cat# HPA030875, RRID:AB_2673640 | WB (1:200) |
| antibody | anti-Mouse IgG (H+L) Highly Cross-Adsorbed Secondary Antibody, Alexa Fluor 647 (Goat polyclonal) | Thermo Fisher Scientific | Cat# A-21236, RRID:AB_2535805 | IF (1:500) |
| recombinant DNA reagent | pet14b containing  coding sequence of SH3 domains of Src, Yes, Fyn, Hck , Lck, Lyn, Fgr and Blk  (Plasmids) | Brian Kay lab  (University of Illinois at Chicago, USA) |  | Used to purify the SH3 domains of SRC family of kinases in this study |
| recombinant DNA reagent | pRK5-c-Fyn (Plasmid) | Addgene | Addgene_16032 |  |
| recombinant DNA reagent | pTriEx-mCerulean-Rac1 (Plasmid) | Klaus Hahn Laboratory (UNC Chapel Hill, USA) |  | mCerulean spectra shown in Figure 3- figure supplement 2C |
| recombinant DNA reagent | pTriEx-mVenus-CBD  (Plasmid) | Klaus Hahn Laboratory (UNC Chapel Hill, USA) |  | mVenus spectra shown in Figure 3- figure supplement 3B |
| recombinant DNA reagent | pRetroSuper-shFyn, shRNA to Fyn kinase  (Plasmid) | Addgene | Addgene_26985 | retroviral  plasmid to  produce retrovirus using AmphoPack-293 as host |
| recombinant DNA reagent | pSUPER retro puro Scr shRNA, scrambled shRNA control  (Plasmid) | Addgene | Addgene_30520 | retroviral  plasmid to  produce retrovirus using AmphoPack-293 as host |
| recombinant DNA reagent | pTriEx-4neo- Novagen  (Plasmid) | Sigma-Aldrich | Cat # 70933 | Used as backbone in this study |
| recombinant DNA reagent | Various constructs used (Plasmids) | This paper |  | A separate list as Supplementary File 2A |
| sequence-based reagent | PCR primers | This paper |  | A separate list as Supplementary File 2B |
| peptide, recombinant protein | Streptavidin, R-Phycoerythrin Conjugate | Thermo Fisher Scientific | Cat # S866 | Flow (1:250) |
| peptide, recombinant protein | PDGF-CC, Human | Sigma-Aldrich | Cat #SPR3139 | Used for Fyn kinase activation in HEK293T and U2OS cells |
| peptide, recombinant protein | PDGF-BB, Mouse | Sigma-Aldrich | Cat #SPR3229 | Used for Fyn kinase activation in C2C12 cells |
| commercial assay or kit | Pierce™ BCA Protein Assay Kit | Thermo Fisher Scientific | Cat # 23225 |  |
| commercial assay or kit | Zymoprep Yeast Plasmid Miniprep II | Zymo-Corp USA | Cat # D2004 |  |
| commercial assay or kit | Glutathione sepharose 4 Fast Flow | Sigma-Aldrich | Cat # GE17-5132-01 |  |
| commercial assay or kit | Ni-NTA agarose beads | Thermo Fisher Scientific | Cat # R90101 |  |
| commercial assay or kit | Dynabeads Biotin Binder | Thermo Fisher Scientific | Cat #11047 |  |
| chemical compound, drug | SU6656 | Sigma-Aldrich | Cat # S9692 | a selective Src family kinase inhibitor used in this paper |
| chemical compound, drug | FAK inhibitor PF-562271 | Selleckchem | Cat# S2890 | FAK inhibitor used in this paper |
| software, algorithm | GraphPad Prism | GraphPad Prism (https://graphpad.com) | RRID:SCR_002798 |  |
| software, algorithm | ImageJ | https://imagej.net/Fiji/ | RRID:SCR_003070 |  |
| software, algorithm | MATLAB | http://www.mathworks.com/products/matlab | RRID:SCR_001622 |  |
| Other | Hoechst 33342 nucleic acid stain | Thermo Fisher Scientific | Cat # H3570 | 10 mg/ml stock , dilution used for IF (1:1000) |
